# De Novo Histoid Leprosy With Unusual Histological Features

**DOI:** 10.7759/cureus.19230

**Published:** 2021-11-03

**Authors:** Fatima Samiey, Jawaher Aljalahma, Ameen Al Awadhi

**Affiliations:** 1 Dermatology, Salmaniya Medical Complex, Manama, BHR

**Keywords:** histoid leprosy, mycobacterium leprae, fite-faraco stain, h&e, de-novo, dapsone, spindle-shaped cells

## Abstract

Histoid leprosy is a type of multibacillary leprosy that has unique clinical and histological characteristics. Some consider it to be an entity of its own, whereas others view it as a variant of lepromatous leprosy. Histoid leprosy can either occur 'de-novo', or secondarily in patients who relapse after dapsone monotherapy or in the presence of dapsone resistance. Here is a case that presented with clinical features of histoid leprosy but also with classic histologic features of lepromatous leprosy, deeming it very distinctive and unusual.

## Introduction

Leprosy, commonly known as Hansen's disease, is a chronic and debilitating condition caused by Mycobacterium leprae and is characterized by granuloma formation in the skin and nerves [1]. It is divided into two polar forms: (1) lepromatous leprosy and (2) tuberculoid leprosy as well as borderline form and an indeterminate form [1].

Given its low incidence as well as its many variants and presentations, it can be challenging to diagnose and is often confused with other granulomatous disorders.

There are only several reports of histoid leprosy in the literature; a rare variant of lepromatous leprosy or sometimes even considered to be its own entity.

## Case presentation

A 37-year-old Indian man not known to have any medical conditions who recently moved to Bahrain was referred to the Dermatology clinic for multiple nodules on the trunk, back, ears, forearms, and thighs. The lesions appeared around one year ago on his thighs, which progressively spread to the rest of his body. The patient had also developed episcleritis a year before the skin lesions started appearing. The patient did not seek any medical care before visiting our clinic, but denied having any other type of skin lesions before the ones he presented with.

**Figure 1:**
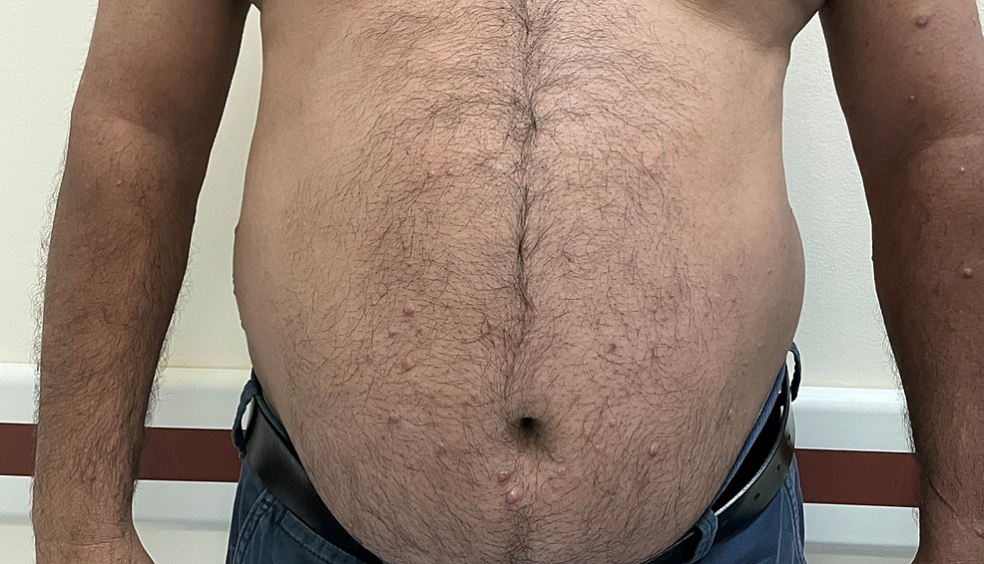
Multiple smooth-surfaced erythematous to skin-colored papules and nodules on the abdomen and arms.

**Figure 2:**
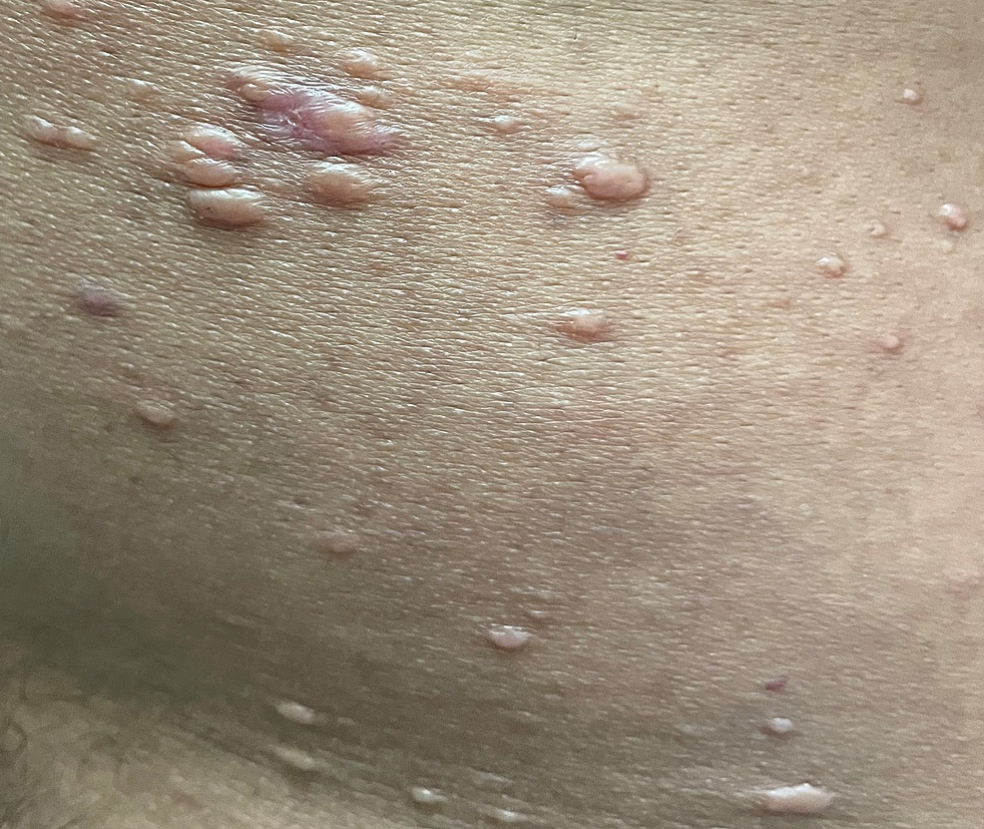
Rubbery skin-colored papules and nodules on the back.

On clinical examination, there were multiple non-tender rubbery erythematous to skin-colored papules and nodules on his abdomen, arms (Figure 1), back (Figure 2) and ears associated with episcleral injection with mild loss of sensation.

The differential diagnoses at the time were: eruptive xanthoma, papular xanthoma, generalized eruptive histiocytoma, cutaneous metastases, multiple leiomyomas and histoid leprosy.

**Figure 3:**
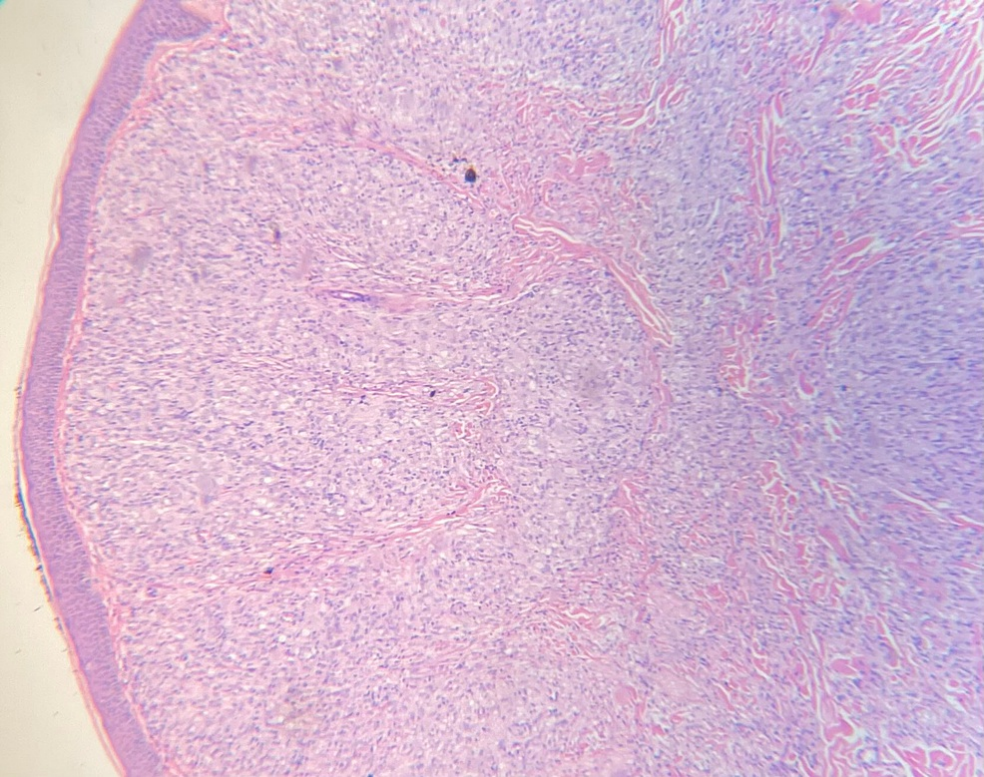
Shave excisional biopsy shows diffuse infiltration of the papillary and reticular dermis by foamy histiocytes admixed with scattered lymphocytes and plasma cells, and a well-formed grenz zone is present between the infiltrate and the epidermis on hematoxylin and eosin stained section (magnification x4).

**Figure 4:**
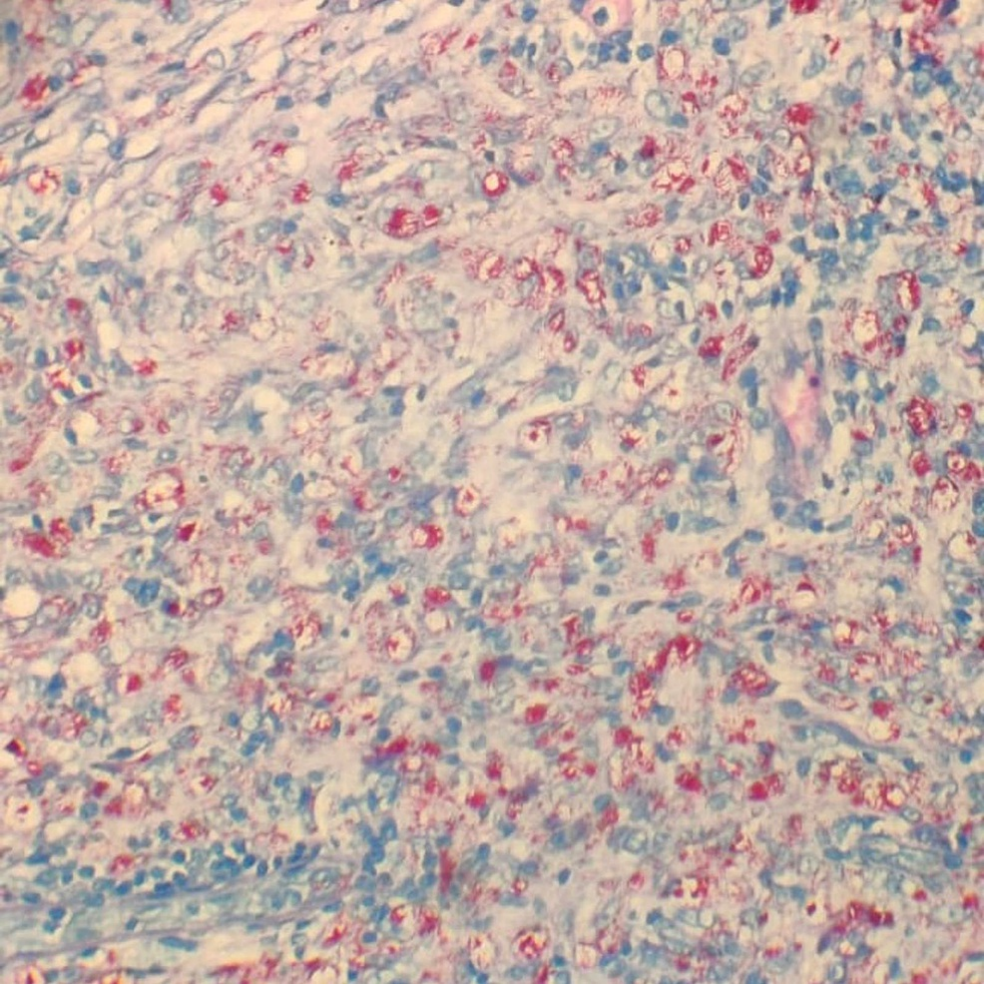
Fite-Faraco stain shows numerous bacilli (pink) (magnification x10).

Two shave biopsies were taken from the back. Hematoxylin and eosin (H&E) sections of the lesions showed diffuse infiltration of the papillary and reticular dermis by foamy histiocytes admixed with scattered lymphocytes and plasma cells, and a well-formed grenz zone was seen between the infiltrate and the epidermis (Figure 3). Fite-Faraco stain showed an abundance of bacilli inside the foamy histiocytes, consistent with a diagnosis of lepromatous leprosy (Figure 4).

Based on clinicopathologic findings, a diagnosis of de-novo histoid leprosy was made. The patient was advised to start multibacillary multidrug therapy.

## Discussion

The term ‘histoid’ was used by Wade to demonstrate the unusual presentation of lepromatous leprosy with distinctive clinical and histopathologic features. The term comes from the entity's characteristic histology, which shows spindle-shaped cells resembling those seen in a dermatofibroma [2].

Histoid leprosy is considered to be a rare variant of lepromatous leprosy by some, and by others a distinct entity. The lesions are often painless, firm, smooth, or umbilicated skin-colored to yellowish-brown papules/nodules on normal-appearing skin [3].

The age at the time of diagnosis is usually between 21 and 40 years, with a predilection towards males [3]. It is often present in patients who have lepromatous leprosy that either relapses after monotherapy treatment with dapsone, in dapsone resistant cases or, rarely, de-novo [3].

The classic histopathological features of histoid leprosy are the circumscribed nature of the lesion, the prevalence of spindle-shaped cells and/or polygonal cells, and an exceptionally large number of acid-fast bacilli as well as absence of globi [4,5]. The bacilli of histoid lesions are noted as being longer than conventional lepra bacilli, making them distinctive. Because of the expanding infiltrate and fibrosis in the dermis, the epidermis overlying the lesion is frequently raised, stretched, and atrophic [4].

The initial management of histoid leprosy is rifampicin 600 mg, ofloxacin 400 mg, and minocycline 200 mg once, followed by multidrug therapy (MDT) for multibacillary (MB) leprosy [3].

The patient presented in this case did not have a previous history of taking dapsone and did not have a background of lepromatous leprosy, thus making this presentation both rare and unusual.

Furthermore, the presence of clinical features of histoid leprosy combined with histologic features of classic lepromatous leprosy is very rare and accounts for a minority of cases of histoid leprosy, making it again a very unique and distinctive presentation.

## Conclusions

Histoid leprosy is considered to be either a rare variant of lepromatous leprosy or perhaps an entity of its own. It is believed to occur either due to dapsone resistance, relapse after monotherapy with dapsone, or de novo. Histopathological features include spindle-shaped cells and a large number of acid-fast bacilli that are longer than the conventional lepra bacilli. Having typical histological features of lepromatous leprosy in histoid leprosy is unusual.
